# Seasonal Rise in the Contents of Microcystin-LR and Odorous Substances Due to Cyanobacterial Blooms in a Drinking Water Reservoir Supplying Xinyang City, China

**DOI:** 10.3390/toxins16100448

**Published:** 2024-10-17

**Authors:** Wei Zhao, Yang Liu, Hua Li, Junguo Ma, Xiaoyu Li

**Affiliations:** 1 College of Life Sciences, Henan Normal University, Xinxiang 453007, China; zhaow1015@163.com (W.Z.); ly@htu.edu.cn (Y.L.); 2 Institute of Hydrobiology, Chinese Academy of Sciences, Wuhan 430072, China; lih@ihb.ac.cn; 3 Henan International Joint Laboratory of Aquatic Toxicology and Health Protection, Henan Normal University, Xinxiang 453007, China; mjunguo_1378@126.com

**Keywords:** drinking water resource, cyanobacterial bloom, microcystin-LR, 2-methylisoborneol, geosmin

## Abstract

Cyanobacterial blooms have become a serious water pollution problem in many parts of the world, and the monitoring and study of the impacts of biotoxins on human health are of vital importance. In this study, the contents of microcystin-LR, 2-methylisoborneol, and geosmin were measured in water and sediment samples from Nanwan Reservoir, China, by means of bimonthly sampling between February and December 2023. The physicochemical and hydrochemical factors and phytoplankton dynamics in the reservoir were also investigated. The results showed that the overall mean concentration of microcystin-LR (0.729 μg/L) in summer approached the guiding standard (1 μg/L) set by the WHO for drinking water. Furthermore, the content of 2-methylisoborneol (143.5 ng/L) was 14 times higher than the national standard (10 ng/L). The results of laboratory cultures showed that lower light levels and medium temperatures were suitable for the growth of *Microcystis* and *Planktothricoides* but higher temperatures promoted the synthesis and release of microcystin-LR and 2-methylisoborneol. In addition, the results of co-cultures showed that the growth of *Planktothricoides* was inhibited by *Microcystis*. Our results suggest that cyanobacterial bloom and the presence of the metabolites 2-methylisoborneol and microcystin-LR can decrease the drinking water quality of Nanwan Reservoir.

## 1. Introduction

Drinking water sources are critically important to the health of urban dwellers. As an important source of drinking water, reservoirs play a vital role in human life. However, in recent years, the general increase in global temperatures, populations, industrialization, and agricultural activities has promoted the rapid eutrophication of rivers, lakes, and reservoirs. As a result, cyanobacterial blooms have frequently occurred in water bodies all over the world [1,2,3,4]. Cyanobacterial blooms produce a large number of harmful secondary metabolites, such as cyanotoxins and taste and odor (T&O) substances, during the growth, metabolism, and death of the cyanobacteria [5,6,7], thereby posing a serious threat to fish, human health, and freshwater ecosystems [8].

Cyanotoxins, especially microcystins (MCs), can directly damage human health. A large number of studies have reported MCs’ toxicity to animals and humans [9,10,11]. However, with the improvement of human living standards, requirements for drinking water quality are becoming stricter; hence, the problem of water odor has begun to attract much attention. In the last decade, many lakes, such as Taihu Lake, Dianchi Lake, Chaohu Lake, and East Lake in China, have exhibited water odor due to heavy cyanobacterial blooms [12,13,14]. This not only leads to public distrust in the quality of drinking water but also poses a certain threat to human health, thus causing serious losses to the fishing and tourism economies.

Nanwan Reservoir is the only source of drinking water for more than six million people in the central urban area of Xinyang City, Henan Province, China. At the end of the last century, due to domestic sewage and agricultural pollution in the upper stream of the reservoir, the water became eutrophicated. Consequently, cyanobacterial blooms have repeatedly occurred in the reservoir; this has caused water pollution and the release of a large number of secondary metabolites (cyanotoxins and odorous substances) into the water body, seriously threatening the stability of the ecosystem and the safety of drinking water for the local residents. Although there have been many studies on the occurrence of cyanobacterial blooms and their influencing factors, the research on the production and influencing factors of cyanotoxins and odors is still very limited, especially on the identification of odor-producing cyanobacteria and the influencing factors [4,7,15,16]. The present study aimed to detect the contents of the main cyanotoxin microcystin-LR (MC-LR) and the odorous substances 2-methylisoborneol (2-MIB) and geosmin in the water and sediment of Nanwan Reservoir by means of bimonthly sampling; additionally, it aimed to explore the possible production mechanisms of cyanotoxins and odorous substances by means of both field investigations and indoor simulation experiments.

## 2. Results

### 2.1. Contents of MC-LR and Odorous Substances Within One Year in the Water and/or Sediment of Nanwan Reservoir

**Figure 1:**
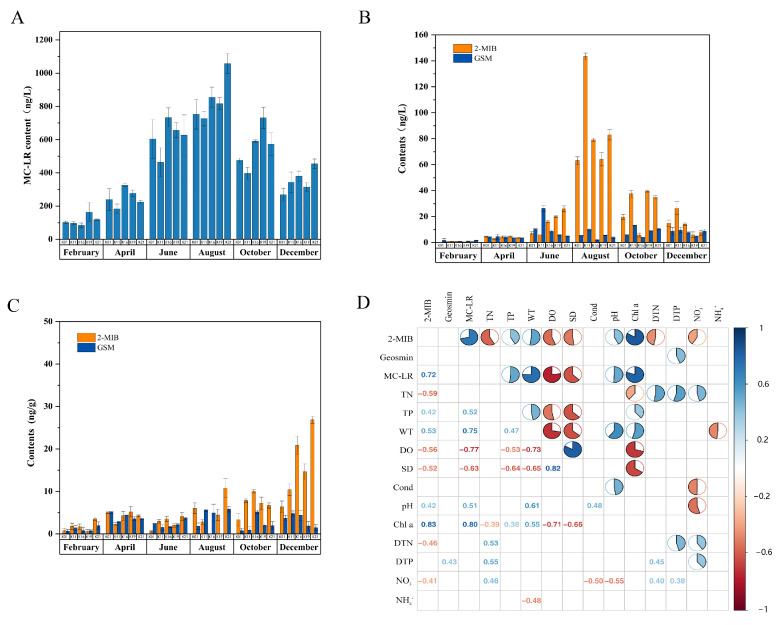
Seasonal concentrations of MC-LR and odorous substances in water and/or sediment from Nanwan Reservoir and the results of a correlation analysis relating odorous substances and toxin with environmental factors. Sampling and contents determination of MC-LR and odorous substances were described in the Section 5 Materials and Methods. The correlation analysis was conducted by using the Corrplot Package of R Language. (**A**) One-year content of MC-LR in the water of Nanwan Reservoir. (**B**) Contents of 2-MIB and geosmin in the water of Nanwan Reservoir. (**C**) Contents of 2-MIB and geosmin in the sediment of Nanwan Reservoir. (**D**) Correlation analysis on the odor substances and toxins with the environmental factors. Blue font indicates positive correlation, red font indicates negative correlation. The number indicates a correlation, and a negative number indicates a negative correlation. Blank space means irrelevant. MC-LR: microcystin-LR; 2-MIB: 2-methylisoborneol; GSM: geosmin.

The concentrations of MC-LR in the water body of Nanwan Reservoir throughout 2023 are shown in Figure 1A; the highest MC-LR concentration was found in summer (1.06 μg/L), while the lowest MC-LR concentration was found at site K16 in February (0.084 μg/L). During the survey period, the MC-LR content at site K21 exceeded the national drinking water safety limit (1 μg/L) only in August, but the contents at all sites were lower than the limit in the remaining months.

In the water body of Nanwan Reservoir, the 2-MIB concentration reached its maximum in summer, with the highest value for the whole year recorded at site K11 in August (143.5 ng/L); this value was 14 times higher than the odor threshold (<10 ng/L) (Figure 1B). The content of geosmin was significantly lower than that of 2-MIB (Figure 1B), with a maximum content of 26.3 ng/L recorded in June. These results suggest that 2-MIB is the main cause of odor in Nanwan Reservoir.

In the sediment from the reservoir, the maximum content of 2-MIB was found in December (26.8 ng/g), while the minimum occurred in June (0.56 ng/g) (Figure 1C), showing a trend of low contents in spring and summer but high contents in autumn and winter. The geosmin content in the sediment was relatively stable throughout the year, with a minimum concentration of 1.12 ng/g recorded in February (Figure 1C).

### 2.2. Effect of Environmental Factors on the Contents of MC-LR and Odorous Substances in the Reservoir

The physical and chemical parameters of the water body throughout 2023 were determined (Figure S1), and a Pearson correlation analysis of these parameters in relation to the 2-MIB, geosmin, and MC-LR contents in one year was carried out. The analysis results showed that the 2-MIB content was positively correlated with TP, WT, pH, and Chl a but negatively correlated with TN, DO, SD, DDN, and NO^3−^. The content of geosmin was positively correlated with DTP but had no significant relationship with any other environmental factor. In addition, the MC-LR content was positively correlated with TP, WT, pH, and Chl a but negatively correlated with DO and SD (Figure 1D).

### 2.3. Algal and Cyanobacterial Composition in the Reservoir and Its Influence on the Contents of MC-LR and Odorous Substances

**Figure 2:**
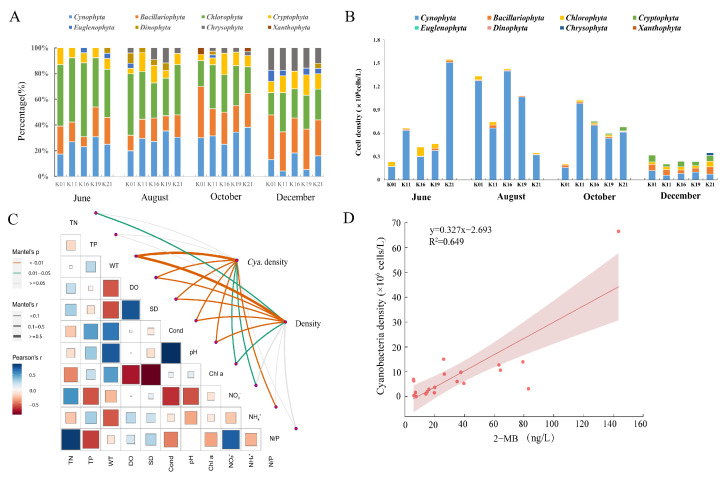
Seasonal alterations in the algal population and cyanobacteria in the water of Nanwan Reservoir and the results of a correlation analysis relating cyanobacteria with environmental factors and 2-MIB contents. Algal identification and biomass assay are described in Section 5.5. The correlation analysis was conducted by using the Mantel Analysis of R Language. (**A**) Seasonal percentage of algal and cyanobacterial composition at various sampling sites. (**B**) Seasonal biomass of algae and cyanobacteria at various sampling sites. (**C**) Correlation analysis on the cyanobacterial density with the environmental factors. Blue font indicates positive correlation, red font indicates negative correlation. The size of the box indicates the correlation level. (**D**) Correlation analysis on the cyanobacterial density with 2-MIB content. 2-MIB: 2-methylisoborneol.

A total of 90 species of algae and cyanobacteria from 59 genera and eight phyla were identified in Nanwan Reservoir (Table S1), among which chlorophytes, diatoms, and cyanobacteria were dominant, as described in Figure 2A,B. In particular, cyanobacteria were dominant in summer and autumn, as determined by their cell density in the range of 1.61 × 10^7^–1.5 × 10^8^ cells/L. However, in winter (December), the cell density of cyanobacteria decreased significantly, reaching a minimum value of 5.91 × 10^6^ cells/L (Figure 2B). The results of a Mantel Test analysis showed that the density of cyanobacteria was significantly positively correlated with WT, DO, SD, Cond, pH, and N:P (*p* < 0.05) (Figure 2C). Meanwhile, a Pearson correlation analysis indicated that the cyanobacterium density was positively correlated with the 2-MIB content (Figure 2D). Concretely, *Planktothricoides* was positively correlated with 2-MIB (*p* < 0.05), *Microcystis* was significantly correlated with the MC-LR concentration, and *Pseudoananais* was positively correlated with geosmin (Table S2).

### 2.4. Morphology and Ultrastructure of the Odor-Producing Cyanobacterium P. raciborskii in Nanwan Reservoir

**Figure 3:**
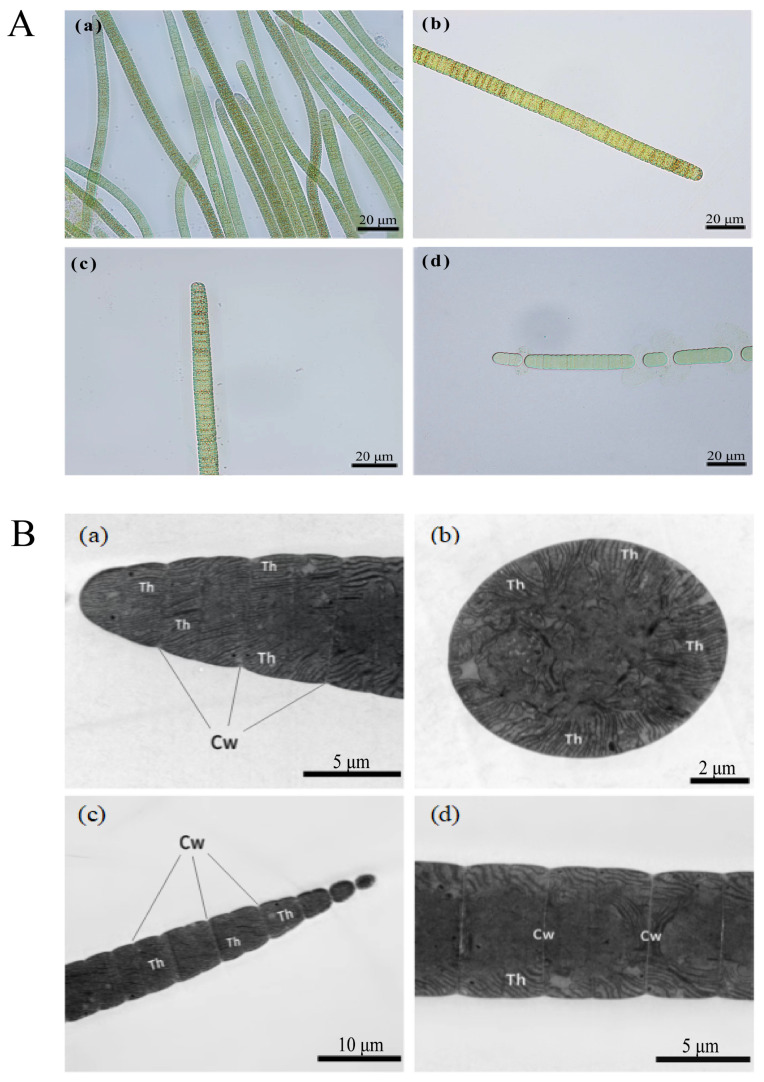
Morphology and ultrastructure of *Planktothricoides raciborskii* isolated from Nanwan Reservoir. (**A**) Morphology of *P. raciborskii* under microscopy. (**a**) Colony filaments; (**b**,**c**) Mono-filaments; (**d**) Dead cells. (**B**) Ultrastructure of *P. raciborskii* by transmission electron microscopy. Cw: cell wall.

Microscopic observations and transmission electron microscopy examinations of *P. raciborskii* showed a solitary filament with air sacs. The filaments were mostly blue-green or yellow-green, and the terminal cells were obtuse or nearly conical, occasionally tapering, without a cap-like structure or sheath. There was no heterocyst or akinete within the filaments. The lengths of the cells were smaller than their widths, with values of 3.6–7 μm and 7.3–13.8 μm, respectively (Figure 3).

### 2.5. Detection of the Odor-Producing Gene Mic and the Microcystin-Producing Gene mcyE via PCR

The microcystin-producing gene (*mcyE*) was detected in *M. aeruginosa*, and the 2-MIB-producing gene (*mic*) was detected in *P. raciborskii* NW-1, but no geosmin synthase gene (*geo*) was detected in the cyanobacteria (Figure S2).

### 2.6. Growth of M. aeruginosa and P. raciborskii and Morphological Alterations in P. raciborskii under Co-Culture Conditions

**Figure 4:**
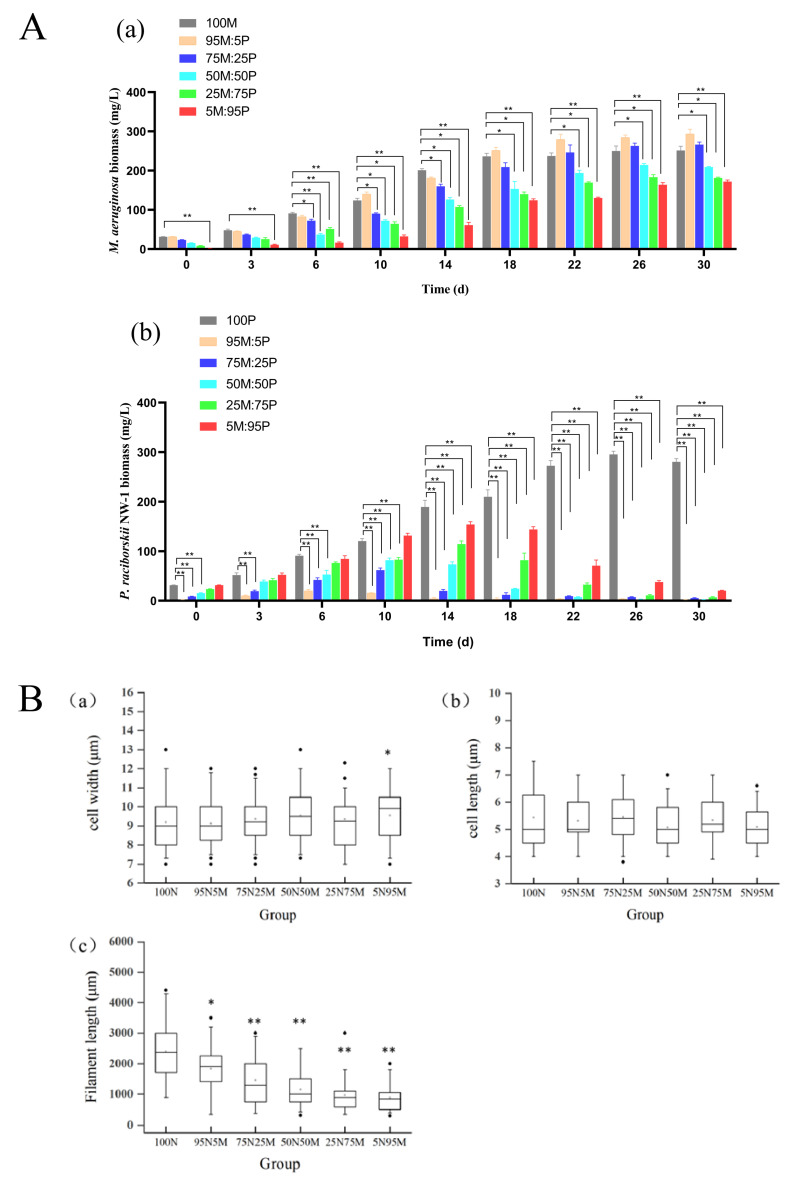
Growth of *Microcystis aeruginosa* and *Planktothricoides raciborskii* and morphological alterations in *P. raciborskii* under co-culture conditions. Biomass assay on *M. aeruginosa* and *P. raciborskii* and morphological measurement on filamentous *P. raciborskii* are described in Section 5.7. (**A**) Growth curves of *M. aeruginosa* and *P. raciborskii*. (**a**) Growth curves of *M. aeruginosa* co-cultured with *P. raciborskii*; (**b**) Growth curves of *P. raciborskii* co-cultured with *M. aeruginosa*. (**B**) Morphology alteration of *P. raciborskii* after co-culture with *M. aeruginosa*. (**a**) Cell width; (**b**) Cell length; (**c**) Filament length. M: *M. aeruginosa*; P: *P. raciborskii*. (* *p* < 0.05; ** *p* < 0.01).

Under laboratory co-culture conditions, *P. raciborskii* had almost no effect on the growth of *M. aeruginosa* (Figure 4A). However, the growth of *P. raciborskii* was significantly suppressed by *M. aeruginosa* (Figure 4A), and the biomass of *P. raciborskii* was almost undetectable in the 95M:5P and 75M:25P groups at the end of the co-culture. Moreover, the filament length of *P. raciborskii* decreased remarkably when it was co-cultured with *M. aeruginosa*, although no obvious alteration in the cellular width or length of *P. raciborskii* was observed (Figure 4B).

### 2.7. Effect of Filtrate Culture on the Growth of M. aeruginosa and P. raciborskii

**Figure 5:**
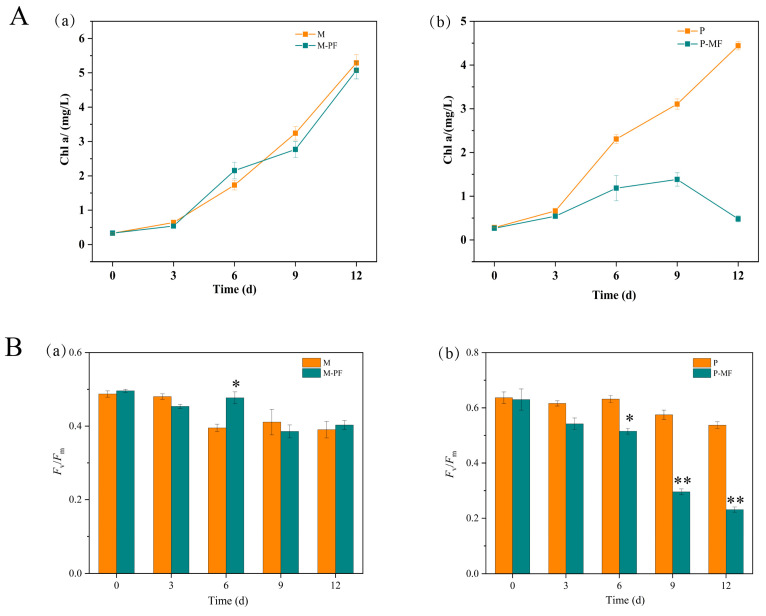
Chlorophyll a contents and photosynthetic fluorescence parameters of *Microcystis aeruginosa* and *Planktothricoides raciborskii* under filtrate culture conditions. The filtrate culture was designed to culture *P. raciborskii*, with the filtrate from the *M. aeruginosa* culture solution obtained by centrifugation to remove *M. aeruginosa* cells or to culture *M. aeruginosa* with the filtrate from the *P. raciborskii* culture solution. Determination of the Chl a content and the photosynthetic fluorescence parameters of the cyanobacteria is described in Section 5.7. (**A**) Chl a content of *Microcystis aeruginosa* (**a**) and *Planktothricoides raciborskii* (**b**); (**B**) *F*_v_/*F*_m_ of *Microcystis aeruginosa* (**a**) and *Planktothricoides raciborskii* (**b**). (* *p* < 0.05; ** *p* < 0.01). *Fv*: Variable Fluorescence; *F*_m_: Maxima Fluorescence. M: *M. aeruginosa*; P: *P. raciborskii*; M-PF: *M. aeruginosa* with filtrate from *P. raciborskii*; P-MF: *P. raciborskii* with the filtrate from *M. aeruginosa*.

The growth of *P. raciborskii* was significantly inhibited after 6 days of culture with filtrate from *M. aeruginosa* (Figure 5A), while no effect of *P. raciborskii* was found on the growth of *M. aeruginosa* (Figure 5B). Furthermore, the photosynthetic fluorescence parameters of *P. raciborskii* were significantly altered under filtrate culture conditions (Figure S3).

### 2.8. Effects of Temperature and Light on the Growth of M. aeruginosa and P. raciborskii and Their Toxin and Odor Production Under Laboratory Conditions

**Figure 6:**
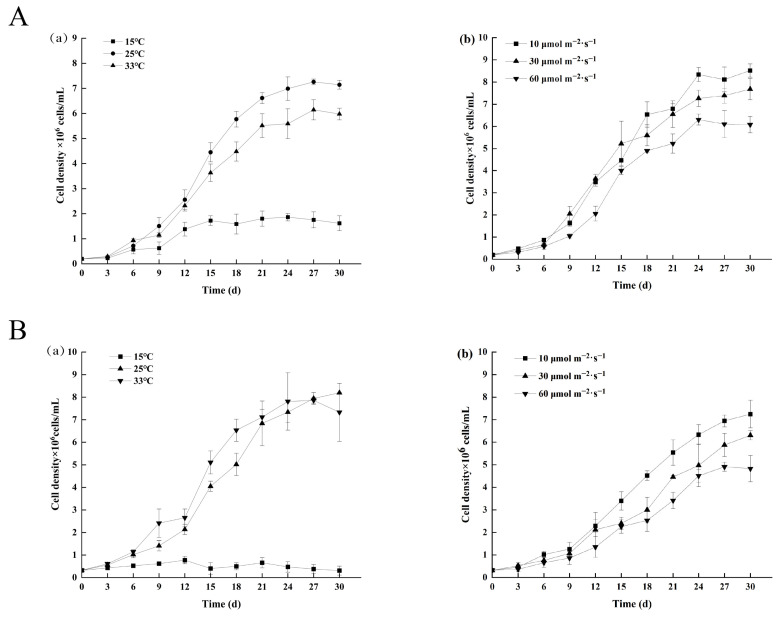
Effects of temperature and light on the growth of *Microcystis aeruginosa* and *Planktothricoides raciborskii* under laboratory conditions. Cyanobacteria culture and biomass assay was described in the Section 5.8. (**A**) Effect of temperature (**a**) and light (**b**) on the growth of *M. aeruginosa*; (**B**) Effect of temperature (**a**) and light (**b**) on the growth of *P. raciborskii*.

**Figure 7:**
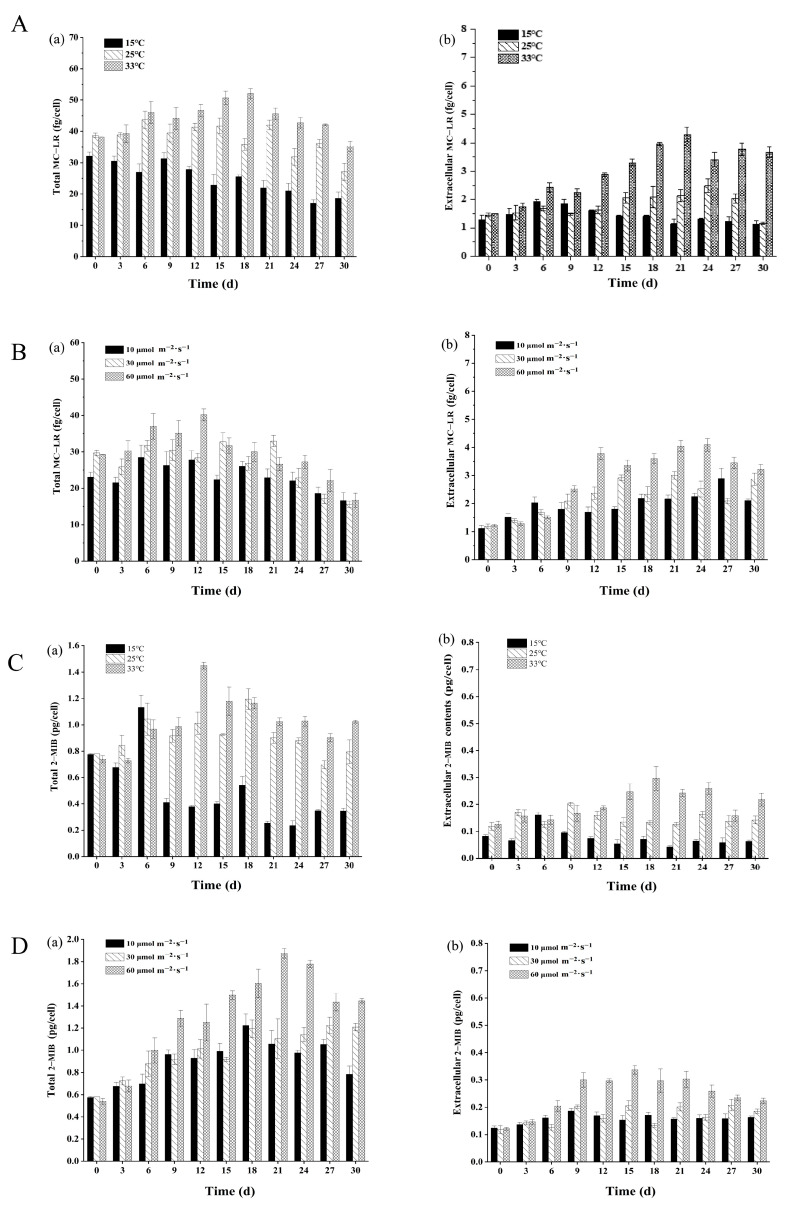
Effects of temperature and light on the production of cyanotoxin and odorous substances by *Microcystis aeruginosa* and *Planktothricoides raciborskii*, respectively. The total MC-LR and extracellular MC-LR from *M. aeruginosa* as well as the contents of total 2-MIB and extracellular 2-MIB from filamentous *P. raciborskii* were measured as described in Section 5.3 and Section 5.4. (**A**) Effects of temperature on the production of the total MC-LR (**a**) and extracellular MC-LR (**b**) from *M. aeruginosa*; (**B**) Effects of light on the production of the total MC-LR (**a**) and extracellular MC-LR (**b**) from *M. aeruginosa*; (**C**) Effects of temperature on the production of the total 2-MIB (**a**) and extracellular 2-MIB (**b**) from *P. raciborskii*; (**D**) Effects of light on the production of the total 2-MIB (**a**) and extracellular 2-MIB (**b**) from *P. raciborskii*. MC-LR: microcystin-LR; 2-MIB: 2-methylisoborneol.

The results of laboratory cultures showed that the optimal growth conditions for *M. aeruginosa* are a temperature of 25 °C and a light intensity of 10 μmol photons *s*^−1^ *m*^−2^ (Figure 6A). MC-LR was found to be mainly present within the cells of *M. aeruginosa*; however, a higher temperature (33 °C) promoted the release of MC-LR from the cyanobacterial cells to the culture solution. Additionally, the highest total MC-LR yield and extracellular MC-LR contents were found in the higher-light-intensity group (60 μmol photons *s*^−1^ *m*^−2^) (Figure 7A,B). For *P. raciborskii*, the optimal growth temperature was 33 °C, and the optimal light intensity was 10 μmol photons *s*^−1^ *m*^−2^ (Figure 6A). It is worth noting that a low temperature (15 °C) completely inhibited the growth of *P. raciborskii*, while higher temperatures (25–33 °C) were favorable for the cellular synthesis and secretion of 2-MIB (Figure 7C). In addition, a high light intensity (60 μmol photons *s*^−1^ *m*^−2^) was more conducive to 2-MIB production (Figure 7D), with maximum total and extracellular contents of (1.87 ± 0.04) pg/cell and (0.33 ± 0.05) pg/cell, respectively.

## 3. Discussion

### 3.1. Seasonal Variations in Contents of MC-LR and Odorous Substances in Nanwan Reservoir

Water eutrophication induces the frequent occurrence of cyanobacterial blooms that then produce harmful substances, posing a threat to water quality and human health. In the present study, testing over the course of one year indicated that the typical cyanotoxin MC-LR and the odorous substances 2-MIB and geosmin were present in Nanwan Reservoir. Moreover, the concentrations of MC-LR and 2-MIB were higher in summer and autumn than in winter and spring, showing significant seasonal characteristics (Figure 1A,B). This result suggests that temperature is a decisive factor affecting the cyanobacterial population. Our subsequent analysis of the correlations between environmental factors and harmful substances from cyanobacteria confirmed this conclusion (Figure 1D). Wu et al. investigated the seasonal dynamics of odorous substance concentrations in Tianmu Lake reservoir and found that the concentration of 2-MIB in the water in summer was positively correlated with the abundance of cyanobacteria [17], which is consistent with the result of this study.

For almost the entire year, the concentration of MC-LR did not exceed the WHO safety standard (1 μg/L) [18], but in summer (from June to August), the MC-LR contents approached the critical point. In the event of future hot and dry seasons, cyanobacterial blooms will become more serious, and the MC-LR limit will be largely exceeded.

Studies have shown that the main sources of odor in lakes and reservoirs are algae and bacteria [19]. However, no geosmin synthase gene (*geo*) was detected in the lab-cultured cyanobacterium *P. raciborskii* (Figure S2), suggesting that geosmin detected in the Reservoir may be produced by other cyanobacteria or actinomycetes. Moreover, numerous studies have shown that cyanobacteria are important producers of 2-MIB [20,21]. In this study, through cyanobacterial investigations and laboratory cultures, it was found that the MC-LR and odorous substances detected in Nanwan Reservoir were produced mainly by *M. aeruginosa* and *P. raciborskii*, respectively. This result indicates that the two dominant cyanobacteria *M. aeruginosa* and *P. raciborskii* may be the main contributors of cyanobacterial toxins and odors in Nanwan Reservoir, representing potential threats to water quality.

### 3.2. Effects of 2-MIB on the Drinking Water Quality of Nanwan Reservoir

A study by Lee et al. showed that 40% of the effluent odor from a water plant was due to the presence of 2-MIB and geosmin [22]. In this study, we found that the 2-MIB content in the water of Nanwan Reservoir reached its highest level in summer, exceeding the olfactory threshold (10 ng/L) by a factor of 14. Subsequently, we investigated the algae and cyanobacteria in the water of Nanwan Reservoir and conducted a correlation analysis to relate the levels of cyanobacteria with environmental factors and 2-MIB contents. The results showed that the concentration of 2-MIB in summer was positively correlated with the abundance of cyanobacteria, especially *P. raciborskii* (Figure 3). Based on this, we isolated the two dominant cyanobacteria, *M. aeruginosa* and *P. raciborskii*, and then cultured them in batches under laboratory conditions. The LC-MS and GC-MS results showed that MC-LR and 2-MIB were produced by *M. aeruginosa* and *P. raciborskii*, respectively. This result suggests that the 2-MIB detected in Nanwan Reservoir might be produced by the filamentous *P. raciborskii*. Su et al. found that *Filamentum* species were the main producers of 2-MIB in a drinking water reservoir in the Yangtze River estuary of China [23]. As Nanwan Reservoir is an important source of drinking water for the local people, the quality and safety of its water are of paramount importance. Therefore, the local drinking water management department should pay close attention to the control of *P. raciborskii* in Nanwan Reservoir.

### 3.3. P. raciborskii in Nanwan Reservoir and the Associated Ecological Risk

Many studies have shown that filamentous *P. raciborskii* is widely distributed in water bodies in Southeast Asia, Africa, and other regions and that it is well-adapted to high temperatures. *P. raciborskii* can produce cyanobacterial bloom in medium- and high-nutrient lakes in summer and autumn, and the secondary metabolites it releases cause the water to have an odor [6,23,24,25]. In this study, 2-MIB-producing *P. raciborskii* was successfully isolated from Nanwan Reservoir and batch-cultured in a laboratory. Through identifications of planktonic algae and biomass assays, it was found that *P. raciborskii* was a dominant species in the reservoir, and GC-MS measurements revealed that it could produce 2-MIB under lab culture conditions. Moreover, we found that it grew well at higher temperatures (30–33 °C) under lab culture conditions; a higher temperature (33 °C) was also beneficial to 2-MIB production. Zhang et al. and Lu et al. studied the effect of temperature on growth and 2-MIB production, and they obtained similar results [6,26]. These studies indicate that the invasive species *P. raciborskii* poses a great threat to the quality of drinking water in Nanwan Reservoir.

### 3.4. The Competitive Relationship Between Two Dominant Cyanobacteria in Nanwan Reservoir

Through this study of phytoplankton in Nanwan Reservoir, two dominant species of cyanobacteria, *M. aeruginosa* and *P. raciborskii*, were identified. We carried out batch cultures of *M. aeruginosa* and *P. raciborskii* in a laboratory, and sufficient cultures were obtained. In order to investigate their ecological relationship, a co-culture of these two kinds of cyanobacteria was then carried out. The results showed that *M. aeruginosa* not only significantly inhibited the growth of *P. raciborskii* but also caused its filaments to become shorter and its cells to become smaller at the later stage of culture, which may be an adaptation to the survival pressure brought about by *M. aeruginosa*. The inhibitory effect of *M. aeruginosa* on the growth of *P. raciborskii* is possibly related to interference with its photosynthesis, but the mechanism is still unclear. There are two possible reasons for this inhibition: one is that *Microcystis* has stronger ability to absorb nutrients than *Planktothricoides*, while another is that *Microcystis* cell division is faster than *Planktothricoides*. The results suggest that *M. aeruginosa* is highly competitive and, therefore, is often the cause of cyanobacterial blooms in natural water bodies.

In addition to differences in light absorption and nutrient utilization between these two dominant species of cyanobacteria, we wanted to know whether their metabolites had an impact on each other’s growth and population survival. Therefore, we carried out an experiment using cyanobacterial filtrate cultures. We found that filtrate from *M. aeruginosa* significantly suppressed the growth of *P. raciborskii*, while filtrate from the latter had little effect on the growth of the former. This result suggests that the metabolites from *M. aeruginosa* can inhibit the growth of *P. raciborskii* but that the specific inhibitory substances, such as allelopathic substance or microcystins, will need to be revealed by further work in the future. Curiously, in natural water bodies such as Nanwan Reservoir, *M. aeruginosa* and *P. raciborskii* can coexist and both become dominant species in the water body, rather than *M. aeruginosa* completely suppressing *P. raciborskii* and causing its population to disappear. This may be due to the fact that there are still some differences in ecological niches between these two dominant cyanobacteria.

## 4. Conclusions

In conclusion, our results showed that the main odorous substance in Nanwan Reservoir was 2-MIB and that, in summer, its content was significantly higher than the olfactory threshold given by the National Standard of China, posing a serious threat to the quality of drinking water in Nanwan Reservoir. *Microcystis aeruginosa* and *Planktothricoides raciborskii* were dominant in summer, and 2-MIB was mainly produced by *P. raciborskii*. The content of MC-LR in summer exceeded or approached the safety standard set by the WHO. Temperature was found to play a decisive role in the growth and metabolite production of *M. aeruginosa* and *P. raciborskii*. In co-culture and filtrate culture experiments, *M. aeruginosa* had an inhibitory effect on the growth of *P. raciborskii*, but further studies are needed to identify the specific metabolic substances involved in this inhibition.

## 5. Materials and Methods

### 5.1. Chemicals

2-Methylisoborneol and geosmin standards (purity ≥ 98%, mother liquor 100 mg/L) were purchased from O2SI, US; a microcystin-LR (MC-LR) standard (≥95%, 1 mg) was purchased from Taiwan Algal Science Inc., China The methanol and acetonitrile used in this study were of chromatographic grade (≥99%, purchased from Sigma Aldrich, St. Louis, MO, USA), and the acetone was analytically pure.

### 5.2. Determination of Physicochemical and Hydrochemical Factors in Nanwan Reservoir

**Figure 8:**
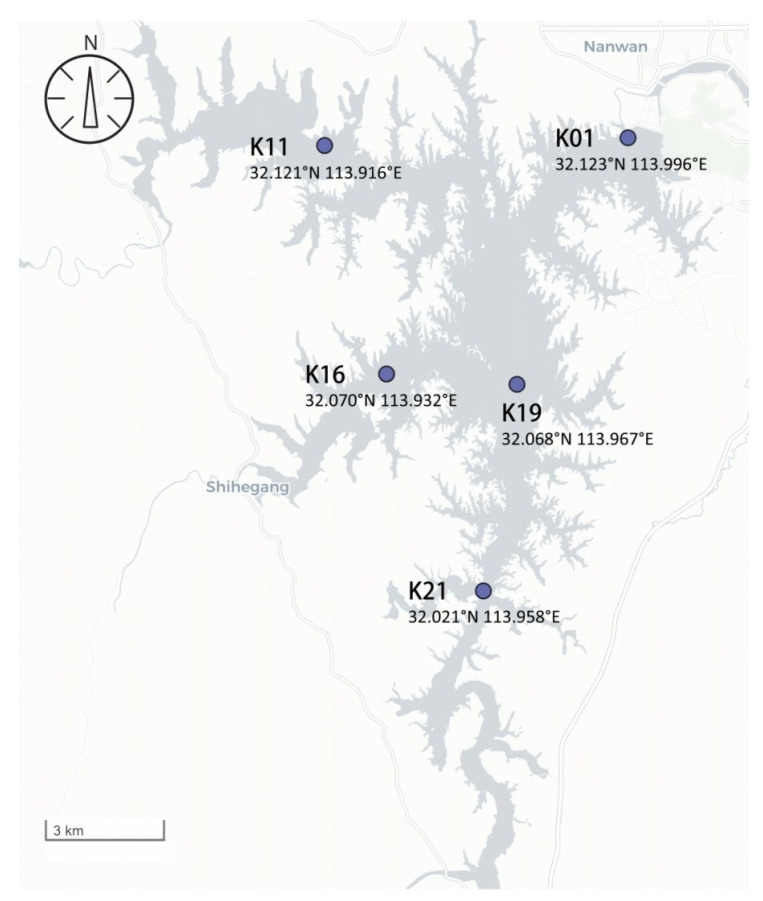
Map of the sampling sites in Nanwan Reservoir.

From February to December 2023, a total of five sampling points were set up in Nanwan Reservoir (31°49′–32°14′ N, 113°45′–114°10′ E) (Figure 8), and the physical and chemical parameters of the water body were detected by means of sampling every other month. The dissolved oxygen (DO), water temperature (WT), pH, and conductivity (Cond) were measured. The water transparency (Secchi depth, SD) was determined using a Secchi disc. A Plexiglass water collector was used to collect water samples at the sampling points; the samples were then refrigerated for transportation to the laboratory. The contents of total nitrogen (TN), total phosphorus (TP), ammonia nitrogen (NH3-N), and nitrate nitrogen (NO3-N) were determined according to the method detailed in Water and Wastewater Monitoring in China [27]. The chlorophyll a content was determined via the method of acetone extraction [28].

### 5.3. Detection of Odorous Substances in Water and Sediment via GC-MS

The contents of odorous substances in water and sediment were detected by using GC-MS according to the methods from the reports by Jeong et al. and Lukassen et al. [29,30]. Water samples were collected 0.5 m below the water surface into 50 mL brown sampling bottles, and three water samples were collected at each sampling site until the bottles were full, without any headspace. Sediment was collected using a Peterson sludge extractor. The samples were packed into brown glass bottles, kept at a low temperature, and protected from light for transport to the laboratory for the immediate detection of odorous substances.

Dried NaCl (1.5 g) was put into a 20 mL brown solid-phase microextraction sample vial, and 5 mL of water sample was added. Then, the vial was tightly capped with a PTFE silicone rubber pad and placed into a sample tray. The tray was transferred to an autosampler shaker and incubated at 65 °C for 10 min at 400 r/min for headspace extraction. After enrichment, the sample was inserted into the injection chamber for thermal desorption for 5 min, and the extraction fibers were aged at 250 °C for 30 min at the time of first use. Odorous substances were detected by using GC-MS (Agilent 7890B/7000C, Agilent, Santa Clara, CA, USA) with the detection limit of 0.25 ng/L for 2-MIB and 0.53 ng/L for geosmin, respectively.

Chromatographic conditions: initial column temperature, 50 °C held for 5 min; temperature raised to 160 °C at 8 °C/min, then raised to 250 °C at 20 °C/min for 4 min; inlet temperature, 250 °C; splitless injection, 1 mL/min.

Mass spectrometry conditions: high-purity helium (99.9% purity); constant pressure, 120 kPa; electron ionization source (EI); ion source temperature, 250 °C; transmission line temperature, 280 °C; ionization energy, 70 eV; scanning mode, select ion scanning monitoring mode (SIM).

### 5.4. Detection of Microcystin-LR via LC-MS

Water samples (1 L) were collected from the sampling sites and transported at low temperature to the laboratory, where the water was filtered through a 0.45 μm GF/C filter. Due to the trace amounts of MCs in samples, sample enrichment was required prior to sample loading. Before each water sample was filtered, the C18 SPE column had to be pre-activated, and then 10 mL of methanol and 10 mL of deionized water were passed through the SPE column in turn, prior to sample loading. After the enrichment, the column was washed with 15 mL of 20% methanol, followed by 10 mL of 100% methanol; finally, the eluent was blown dry with a nitrogen blowing instrument, the volume was fixed to 1 mL with methanol, and the sample was filtered through a 0.22 μm organic filter membrane before the assay. Microcystin-LR was detected by using LC-MS (ACQUITY UPLC H-class-Xevo TQ MS, Waters, Milford, MA, USA) with the detection limit of 20 ng/L according to the method from the report by Neffling et al. [31].

Chromatographic conditions: column, ACQUITY UPLC^®^BEH C18 (1.7 μm, 2.1 × 50 mm); temperature, 45 °C; flow, 0.4 mL/min; injection volume, 10 μL; mobile phase A, 0.1% formic acid (aqueous) solution; mobile phase B, acetonitrile.

Mass spectrometry conditions: electrospray ion source (ESI); positive ion scanning; multiple reaction monitoring (MRM) mode; capillary voltage, 2.5 kV; ion source temperature, 150 °C; desolubilization gas temperature, 500 °C; desolubilization gas flow rate, 900 L/h; cone gas flow rate, 50 L/h.

### 5.5. Identification of Planktonic Algae and Cyanobacteria and Biomass Assay

Water samples for algal and cyanobacterial identification were taken using a No. 25 plankton net (64 μm). The concentrated algal and cyanobacteria samples were transferred to sampling bottles and fixed with 4% formaldehyde. Algal and cyanobacterial identification was conducted according to “Freshwater Plankton Research Methods” [28].

The water samples (1 L) for algal and cyanobacteria biomass assays were taken at each sampling site and fixed with 4% formaldehyde. After the samples were left to stand for 48 h, the upper liquid was removed. The samples were concentrated to 50 mL and sealed in the dark. The number of algae and cyanobacteria was then determined using a planktonic counting chamber under a microscope.

### 5.6. Laboratory Culture of Microcystis aeruginosa and Planktothricoides raciborskii from the Reservoir and Expression Analysis of Their Toxin-Producing and Odor-Producing Genes

Cyanobacteria were isolated using a Pasteur capillary, and each obtained single cyanobacterial cell or filament was inserted into a 24-well cell culture plate filled with BG11 medium [28]. The plate was sealed with parafilm and placed in a light incubator with a temperature of 25 ± 0.5 °C, a light intensity of 25 μmol photons *s*^−1^ *m*^−2^, and a light-to-dark ratio of 12 h:12 h. After about 15 days of incubation, each single cyanobacterial cell or filament was transferred to a glass test tube under sterile conditions for seed preservation; this transfer was carried out regularly.

Polymerase chain reaction (PCR) amplification was performed to determine whether the two strains of cyanobacteria contained odor-producing cyclase genes (*mic*), geosmin genes (*geo*), or microcystin genes (*mcyE*), using the primers listed in Table S3 [29,30,32]. The PCR was conducted in a 50 μL reaction system including 2 × U Taq MasterMix 25 μL, 1 μL of 10 μmol/L upstream and downstream primers each, 2 μL of DNA template, and ddH_2_O complement to 50 μL. The PCR reaction conditions were: pre-deformation at 94 °C for 10 min; denatured at 94 °C for 30 s, annealed at 56 °C for 30 s; extended at 72 °C for 45 s, 35 cycles in this stage. The PCR products were examined by 1% agarose gel electrophoresis to ensure that the amplified sequences were obtained.

### 5.7. Co-Culture and Filtrate Culture of Microcystis aeruginosa and Planktothricoides raciborskii

The two dominant cyanobacteria *M. aeruginosa* (M) and *P. raciborskii* (P) from Nanwan Reservoir were co-cultured according to the method used by Omidi et al. to evaluate their relationship in terms of population growth [33]. The experiment was divided into six groups: 100M = 100% *M. aeruginosa*; 95M:5P = 95% *M. aeruginosa* + 5% *P. raciborskii*; 75M:25P = 75% *M. aeruginosa* + 25% *P. raciborskii*; and so on. Each group contained the above cyanobacteria (*M* and *P*) at different ratios for co-culture in three replicates. The two kinds of cyanobacteria in the logarithmic growth phase were transferred to the corresponding groups according to the pre-designed ratios; the initial total biomass of cyanobacteria in each group was 32 μg/L. The culture lasted for 30 days, and samples were taken on the 0th, 3rd, 6th, 10th, 14th, 18th, 22nd, 26th, and 30th days. A 5 mL volume of culture solution was taken out each time and fixed with a 1% volume of 4% formaldehyde; the fixative solution was shaken well prior to cyanobacteria counting using a phytoplankton counting chamber under a microscope (Olympus, Tokyo, Japan). Due to the large difference in cell volume between the two strains of cyanobacteria, the cell density was uniformly converted into biomass according to the calculation method detailed by Hillebrand et al. [34]. On the 22nd day of culture, a morphological analysis of each experimental group of *P. raciborskii* was carried out, including measurements of the cell length and width and the length of filaments. At least 200 cells and 150 filaments were randomly selected for measurement.

The filtrate culture experiment in this study was designed to culture *P. raciborskii* with filtrate from an *M. aeruginosa* culture solution (obtained via centrifugation to remove the *M. aeruginosa* cells) and to culture *M. aeruginosa* with filtrate from a *P. raciborskii* culture solution. The filtrate was obtained by means of the low-speed centrifugation of the culture solution (4000× *g*, 8 min) to remove *M. aeruginosa* cells or *P. raciborskii* filaments in the logarithmic growth phase, followed by the suction filtration of the supernatant (0.45 μm, Whatman GF/C filter). The concentrations of TN and TP in the filtrate culture were consistent with those in the BG11 medium to guarantee that the growth of the cyanobacteria was not restricted by nutrients.

The chlorophyll a (Chl a) content in the culture solution was determined via the method of acetone extraction [28]. Briefly, 5 mL of cyanobacterial culture solution was taken and centrifuged at 4500× *g* for 8 min. After centrifugation, the supernatant was removed, and 5 mL 90% acetone was added. The solution was placed in the dark at 4 °C for 24 h, then centrifuged again at 8000× *g* for 15 min, and the supernatant was transferred into a quartz cuvette. The light absorption values at 750, 663, 645, and 630 nm were determined using a UV landscape photometer.

The photosynthetic fluorescence parameters (the maximum photosynthetic efficiency (Fv/Fm), the maximum electron transfer rate (ETRmax), the initial slope of the photosynthetic curve (alpha), and the semi-saturated light intensity (IK) of the photosynthetic system II (PS II)) of algae in each group were detected every 3 days using a Dual-PAM two-channel phytoplankton fluorometer.

### 5.8. Growth Characteristics of M. aeruginosa and P. raciborskii and Their Cyanotoxin or Odorous Substance Production in Laboratory Cultures at Different Temperatures and Light Intensities

*M aeruginosa* and *P. raciborskii* were inoculated into BG11 medium in a shaker with a light intensity of 25 μmol photons *s*^−1^ *m*^−2^ at 25 °C and a light-to-dark ratio of 12 h:12 h (L:D). The cyanobacteria were collected in the logarithmic growth phase by means of centrifugation and rinsed several times with sterile water, then inoculated again in BG11 medium with initial inoculation densities of 3 × 10^5^ cells/mL for *P. raciborskii* and 2 × 10^6^ cells/mL for *M. aeruginosa*. For the temperature treatment group, three temperatures (15, 25, 33 °C) were selected at a light intensity of 25 μmol photons *s^−1^ m^−2^*, while for the light treatment group, the light intensity was set to 10, 30, or 60 μmol photons *s*^−1^ *m*^−2^, and the temperature was kept at 25 °C. Three parallel samples were set up in each group. The growth determination of *M. aeruginosa* and *P. raciborskii* was conducted at 3-day intervals according to the method described in Section 5.7. The total 2-MIB and extracellular 2-MIB contents from filamentous *P. raciborskii* cyanobacteria, as well as the total MC-LR and extracellular MC-LR contents from *M. aeruginosa*, were also measured at 3-day intervals using the methods described in Section 5.3 and Section 5.4.

### 5.9. Statistical Analysis

All statistical analyses were performed using GraphPad Prism 8.00 (San Diego, CA, USA) and SPSS 25.0 (Chicago, IL, USA). A value of *p* < 0.05 was considered to indicate a significant difference between the treatment group and the control group in pairwise analyses via one-way ANOVA or t-test; such differences are indicated by asterisks in the figures (* *p* < 0.05; ** *p* < 0.01).

## Data Availability

Data will be made available on request.

## Supplementary Materials

The following supporting information can be downloaded at: https://www.mdpi.com/article/10.3390/toxins16100448/s1. Table S1: Algae identified in Nanwan Reservoir; Table S2: Pearson correlation analysis relating MC-LR and odorous substances with the dominant cyanobacteria from Nanwan Reservoir; Table S3: Primer sequences for synthetic genes; Figure S1: Physicochemical and hydrochemical parameters of the water body of Nanwan Reservoir throughout 2023; Figure S2: Amplification electropherogram of the 2-MIB-producing gene *mic* and the microcystin-producing gene *mcyE*. Figure S3: Photosynthetic fluorescence parameters of *Microcystis aeruginosa* and *Planktothricoides raciborskii* under filtrate culture conditions.
