# Supplementary material for: Seasonal Rise in the Contents of Microcystin-LR and Odorous Substances Due to Cyanobacterial Blooms in a Drinking Water Reservoir Supplying Xinyang City, China
Source: Toxins (Basel). 2024 Oct 17;16(10):448. doi: 10.3390/toxins16100448 (PMC11511344; doi:10.3390/toxins16100448)
Supplement: Supplementary file 1 [file toxins-16-00448-s001.zip › toxins-3200454-supplementary.pdf]

Supplementary materials

Table S1 Algae and cyanobacteria identified in Nanwan Reservoir

	**Cynophyta**
1	*Microcystis* sp.
2	*Microcystis wesenbergii*
3	*Chroococcus* sp.
4	*Merismopedia* sp.
5	*Limnolyngbya circumcreta*
6	*Leptolyngbya* sp.
7	*Pseudoanabaena* sp.
8	*Aphanocapsa* sp.
9	*Rhabdogloea* sp.
10	*Dactylococcopsis* sp.
11	*Planktothricoides* sp.
12	*Oscillatoria* sp.
13	*Oscillatoria princeps*
14	*Limnothrix* sp.
15	*Aphanizomenon* sp.
16	*Cuspidothrix issatschenkoi*
17	*Dolichospermum* sp.
18	*Cylindrospermopsis* sp.
19	*Raphidiopsis* sp.
	**Bacillariophyta**
1	*Melosira* sp.
2	*Melosira granulata*
3	*Cyclotella* sp.
4	*Fragilaria* sp.
5	*Synedra* sp.
6	Achnanthes sp.
7	*Cocconeis* sp.
8	*Rhizosolenia* sp.
9	*Attheya* sp.
10	*Asterionella glacialis*
11	*Navicula* sp.
12	*Gomphonema* sp.
13	*Nitzschia* sp.
	**Chlorophyta**
1	*Chlamydomonas* sp.

2	*Coccomonas orbicularis*
3	*Planktosphaeria gelatinosa*
4	*Elakatothrix gelatinosa*
5	*Golenkinia* sp.
6	*Schroederia* sp.
7	*Chodatella wratislaviensis*
8	*Chodatella ciliata*
9	*Tetraëdron minimum*
10	*Selenastrum* sp.
11	*Sphaercystis* sp.
12	*Oocystis* sp.
13	*Dictyosphaerium* sp.
14	*Dictyosphaerium pulchellum*
15	*Pediastrum simplex var. duodenarium*
16	*Scenedesmus* sp.
17	*Scenedesmus quadricauda*
18	*Crucigenia apiculata*
19	*Actinastrum* sp.
20	*Coelastrum* sp.
21	*Coelastrum reticulatum*
22	*Closterium* sp.
23	*Cosmarium* sp.
24	*Chlorogonium* sp.
25	*Micractinium* sp.
26	*Schroederia nitzschioides*
27	*Chodatella* sp.
28	*Chodatella longiseta*
29	*Tetraëdron caudatum*
30	*Ankistrodesmus acicularis*
31	*Kirchneriella* sp.
32	*Treubaria crassispina*
33	*Pediastrum duplex var.gracillimum*
34	*Crucigenia tetrapedia*
35	*Asterococcus superbus*
36	*Chlorococcum* sp.
37	*Characium* sp.
38	*Tetraëdron minimum*
39	*Ankistrodesmus* sp.
40	*Crucigenia quadrata*
41	*Mougeotia* sp.

42	*Staurastrum* sp.
	**Cryptophyta**
1	*Chroomonas* sp.
2	*Chroomonas acuta*
3	*Cryptomonas* sp.
4	*Cryptomonas ovata*
5	*Cryptomonas erosa*
6	*Cryptomonas rostrata*
	**Euglenophyta**
1	*Euglena* sp.
	**Dinophyta**
1	*Peridinium* sp.
2	*Gymnodinium* sp.
	**Chrysophyta**
1	*Chrysococcus* sp.
2	*Mallomonas* sp.
3	*Synura* sp.
4	*Chromulina* sp.
5	*Dinobryon* sp.
6	*Ochromonas* sp.
7	*Chrysococcus* sp.

Table S2 Pearson correlation analysis on MC-LR and odorous substances with the dominant cyanobacteria from Nanwan Reservoir

	Mic.	*Pse.*	*Plank.*	*Dol.*	*Cyl.*	*Frag.*
2-MIB	-	-	0.81	0.47	0.42	0.46
Geosmin	-	0.41	-	-	-	-
MC-LR	0.73	-	0.56	0.49	0.52	-

MC-LR: microcystin-LR. 2-MIB: 2-methylisoborneol.

Mic: *Microcystis* sp. *Pse*: *Pseudoanabaena* sp. *Plank*: *Planktothricoides* sp. *Dol*: *Dolichospermum* sp. *Cyl*: *Cylindrospermopsis* sp. *Frag*: *Fragilaria* sp.

Table S3 Primer sequences for synthetic genes

**Primers**	**Sequences**	**Products**	**Target fragment length (bp)**
MIB-Rf	CGACAGCTTCTACAYCYCCATGAC	2-MIB	202
MIB-Rr	CGCCGCAATCTGTAGCACCAT		
*geo*f	TGGTATGTNTGGGTRTTCTT	geosmin	311
*geo*Ir	ATGTATTCRATGGGGTTRGC		
mcyE-F2	GAAATTTGTGTAGAAGGTGC	mcyE	812
mcyE-R4	AATTCTAAAGCCCAAAGACG		

2-MIB: 2-methylisoborneol.

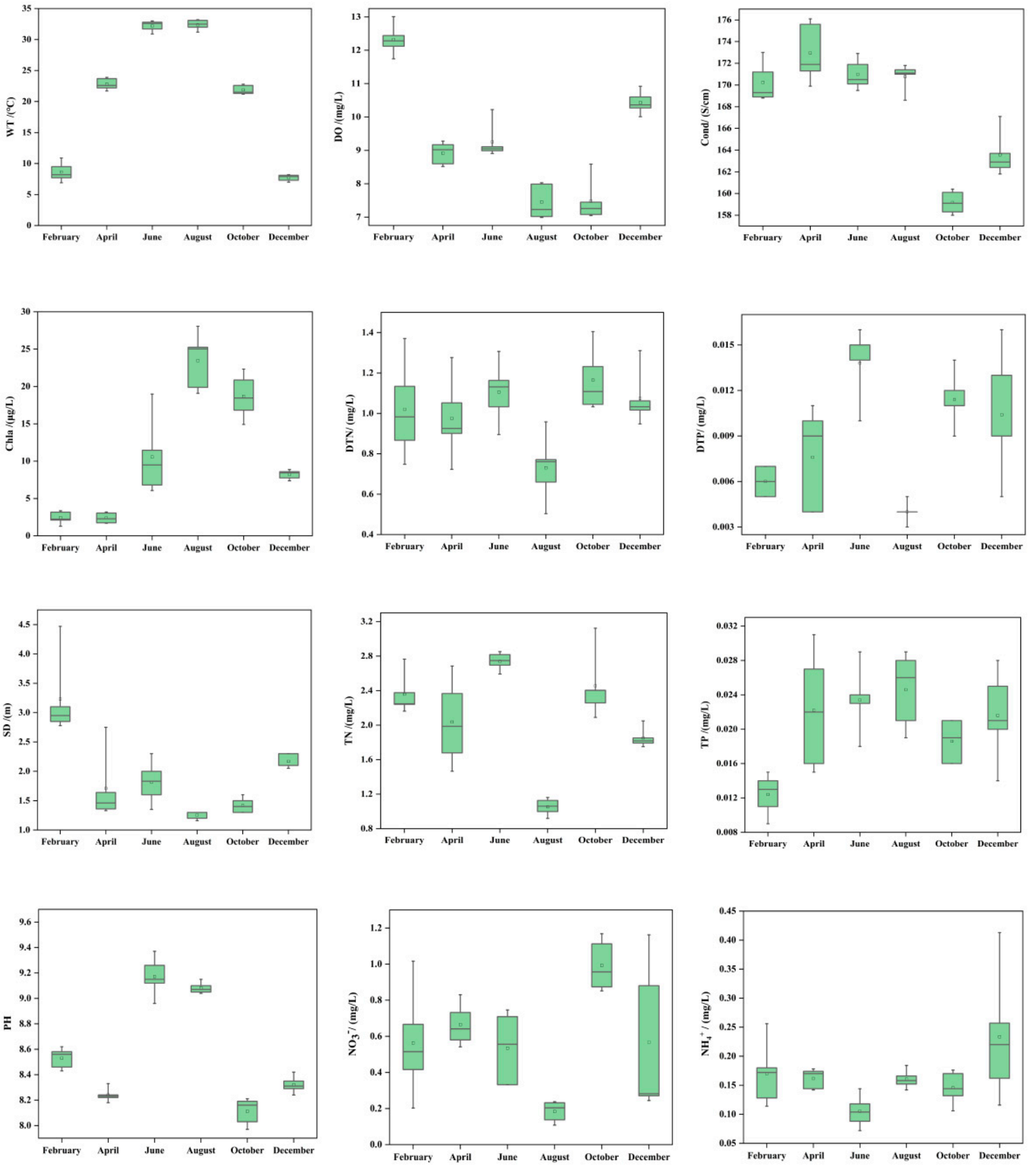

Figure S1 Physicochemical and hydrochemical parameters in the water body of Nanwan Reservoir throughout 2023.

The physical and chemical factors of the water body were detected by sampling every other month according to the determination method in Water and Wastewater Monitoring in China (Fourth Edition) (2002).

DO: dissolved oxygen; WT: water temperature; Cond: conductivity; Chl a: chlorophyll a; SD: Secchi depth. TN: total nitrogen; TP: total phosphorus; NH^3-N: ammonia nitrogen; NO^3-N: nitrate nitrogen.

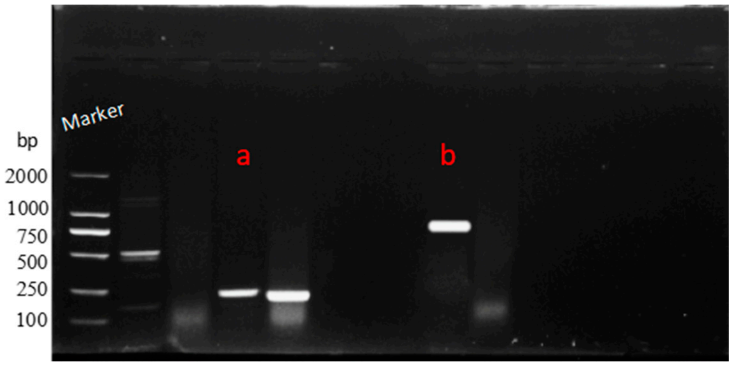

Figure S2 Amplification electropherogram of the 2-MIB-producing gene *mic* and microcystin-producing gene *mcyE*.

a: *Planktothricoides raciborskii*; b: *Microcystis aeruginosa*; 2-MIB: 2-methylisoborneol.

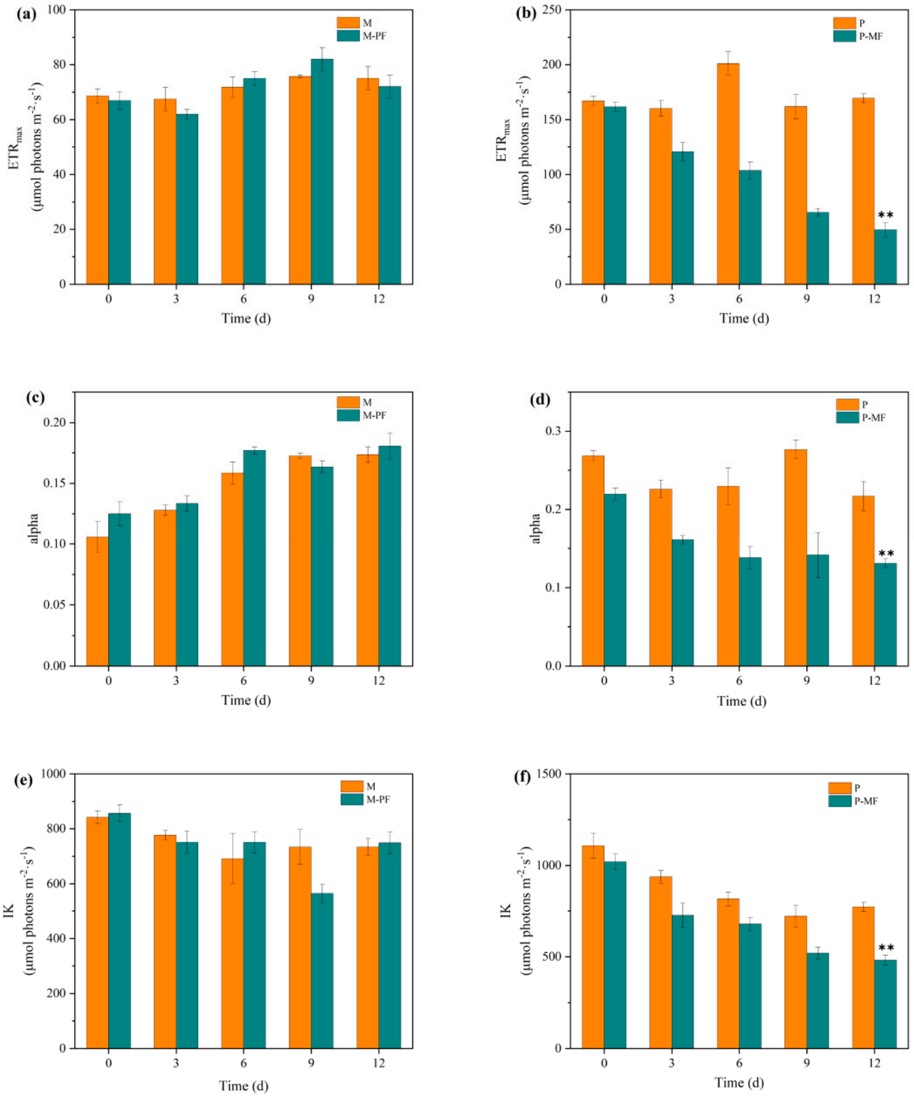

Figure S3 a-b: ETRmax; c-d: alpha; e-f: IK; (**p* < 0.05; ** *p* < 0.01).
ETRmax: The maximum electron transfer rate; alpha: Initial slope of Light Curve;
IK: Half-Saturation Light Intensity.
